# To treat or not to treat? Adjuvant chemotherapy in 46 patients with stage IB lung squamous cell carcinoma: a real-world matched analysis

**DOI:** 10.3389/fonc.2025.1647649

**Published:** 2025-10-08

**Authors:** Botao Liang, Tingting Fu, Juntang Guo, Lianbin Zhang, Chaoyang Liang, Tao Zhang, Kai Zhao, Zhaofeng Gao, Jing Yang, Leilei Shen

**Affiliations:** ^1^ Department of Intensive Care Unit, Traditional Chinese and Western Medicine Hospital, Tianshui, China; ^2^ Department of Anesthesia and Nursing Center, First Medical Center of People's Liberation Army General Hospital, Beijing, China; ^3^ Department of Thoracic Surgery, First Medical Center of People's Liberation Army General Hospital, Beijing, China; ^4^ Department of Thoracic Surgery, Hainan Hospital of People's Liberation Army General Hospital, Sanya, China; ^5^ School of Medicine, Nankai University, Tianjin, China

**Keywords:** adjuvant platinum-based chemotherapy, adjuvant therapy, squamous cell carcinoma, stage IB, overall survival, disease-free survival

## Abstract

**Background:**

The value of adjuvant platinum-based chemotherapy (ACT) in stage IB squamous cell carcinoma (SqCC) remains controversial.

**Methods:**

This retrospective study enrolled patients with surgically resected stage IB SqCC between January 2013 and August 2024. Clinicopathological characteristics and survival data were collected. To mitigate the impact of observed confounders, propensity score matching (PSM) was performed using clinically relevant covariates, including age, sex, tumor diameter, poor differentiation, spread through air spaces, visceral pleural invasion, and lymphovascular invasion, resulting in 46 well-matched patient pairs. The primary endpoint was disease-free survival (DFS). The secondary endpoint was overall survival (OS).

**Results:**

A total of 181 patients were enrolled in the study. Among them, 50 patients (27.6%) received adjuvant chemotherapy (ACT), while 131 patients (72.4%) were assigned to the clinical observation (CO) group. The ACT group had more patients <65 years (*P* = 0.007), larger tumors (*P* < 0.001), and more poorly differentiated tumors (*P* = 0.017) compared with the CO group. The two groups were comparable after PSM. Among the ACT group, twenty-eight (56%) patients received cisplatin plus docetaxel regimen. 16% (8/50) of patients took cisplatin plus gemcitabine regimen, and carboplatin plus paclitaxel was 14 (28%) patients’ option. The median follow-up time was 60 months (range: 5–137 months). The 5-year DFS rates were 84.8% in the ACT group and 85.6% in the CO group (HR: 1.05, 95% CI 0.44-2.51; *P* = 0.919). There was no statistically significant difference in DFS between groups, both in the entire cohort (*P* = 0.701) and the matched cohort (*P* = 0.665). The 5-year OS rates were 96.8% in the ACT group and 93.1% in the CO group (HR: 2.46, 95% CI 0.72-8.35; *P* = 0.150). No statistically significant difference in OS was observed, either in the overall cohort (*P* = 0.131) or in the matched cohort (*P* = 0.300).

**Conclusions:**

ACT may not confer significant survival benefits in resected stage IB SqCC, with comparable disease-free and overall survival outcomes observed between treatment and observation groups.

## 1 Introduction

Lobectomy combined with systematic nodal dissection remains the standard treatment for early-stage non-small cell lung cancer (NSCLC) (1). However, only 16% of patients achieve a stage I diagnosis following radical resection (2). Notably, even among these stage I patients, those with stage IB disease demonstrate a 5-year overall survival (OS) rate of merely 69%, indicating that approximately one-third of patients will ultimately experience disease recurrence, metastasis, and cancer-related mortality (3). Undetectable minimal residual disease (MRD) may contribute to disease relapse, highlighting the critical need for MRD eradication through adjuvant therapy (4). However, previous studies have failed to demonstrate a significant survival benefit from platinum-based adjuvant chemotherapy in stage IB NSCLC (5–11). Current clinical guidelines, including those from the European Society for Medical Oncology (ESMO) and the American Society of Clinical Oncology (ASCO), do not recommend adjuvant platinum-based chemotherapy (ACT) for completely resected stage IB NSCLC (12, 13). Notably, only the National Comprehensive Cancer Network (NCCN) guideline suggests considering ACT or epidermal growth factor receptor-tyrosine kinase inhibitors (EGFR-TKIs) for high-risk stage IB patients (14). Recent landmark studies, including the ADAURA trial (15), CORIN study (16), and research by Shen et al. (17), have demonstrated that adjuvant EGFR-TKIs significantly improve disease-free survival (DFS) compared to observation in stage IB lung adenocarcinoma patients harboring EGFR mutations. Notably, the DFS benefit with adjuvant osimertinib translated into a remarkable overall survival (OS) advantage, achieving an unprecedented 5-year OS rate of 94% (15).

However, limited evidence exists regarding the efficacy of adjuvant treatment for stage IB pulmonary squamous cell carcinoma (SqCC). To address this knowledge gap, we conducted a real-world comparative study evaluating ACT versus observation in patients with completely resected stage IB SqCC.

## 2 Patients and methods

### 2.1 Patient selection and study design

We conducted a retrospective review of patients treated at our thoracic surgery center between January 2013 and August 2024. Inclusion criteria required: (1) age ≥18 years; (2) ECOG performance status 0-1; (3) completely resected stage IB (T2aN0M0) SqCC according to UICC/AJCC 9^th^ edition staging criteria (3); and (4) completion of ≥2 cycles of platinum-based doublet chemotherapy (without immunotherapy) initiated 4–8 weeks postoperatively. Eligible surgical procedures included lobectomy, sleeve lobectomy, bilobectomy, or pneumonectomy with systematic lymph node dissection. Exclusion criteria comprised: (1) preinvasive or minimally invasive SqCC; (2) metastatic disease; (3) receipt of neoadjuvant therapy; (4) perioperative mortality (death within 30 days post-surgery); (5) protocol non-compliance; or (6) loss to follow-up.

**Figure 1:**
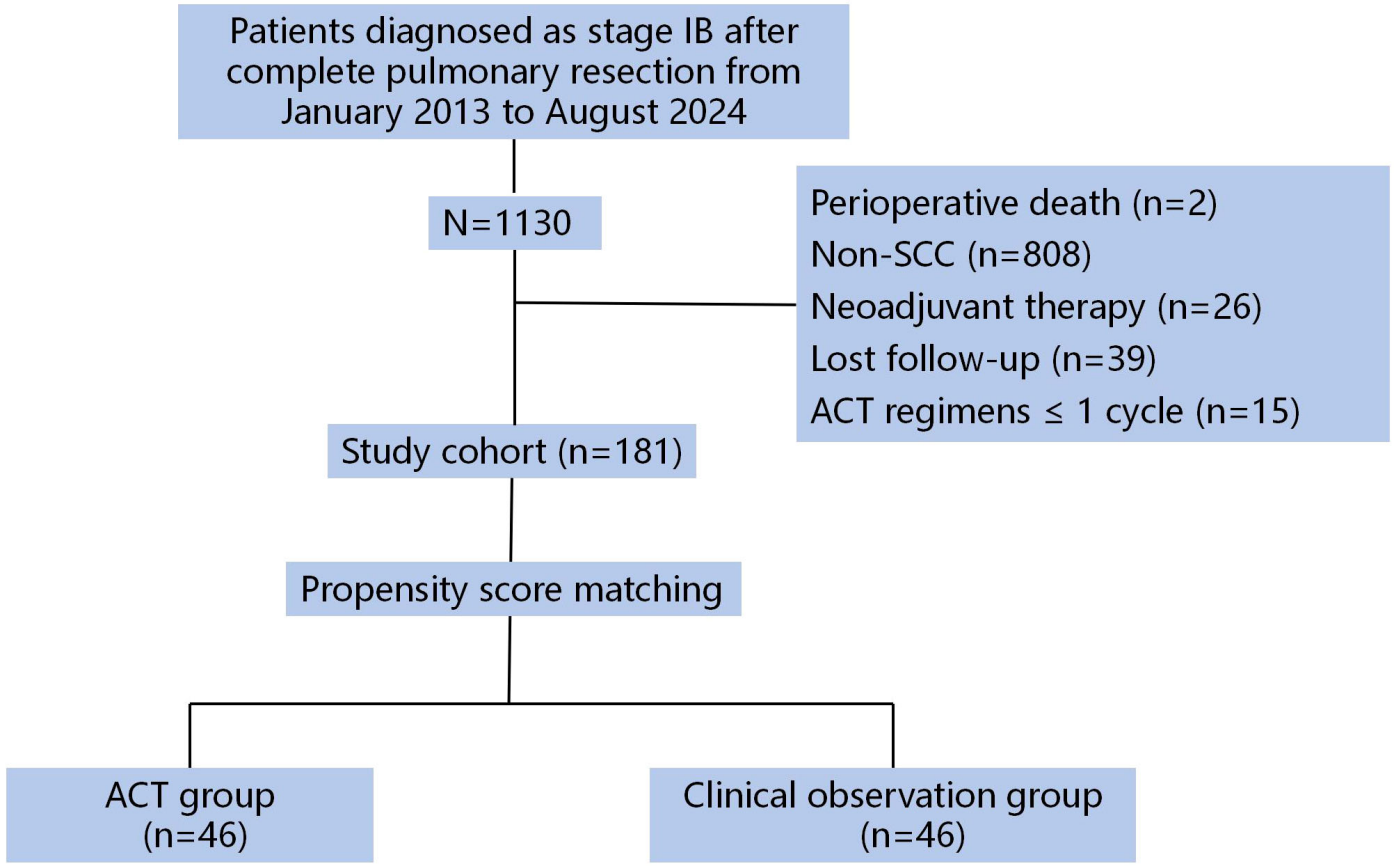
Study enrollment flow diagram.

From an initial pool of 1130 screened patients, 181 met all eligibility criteria (**Figure 1**). Clinicopathological characteristics and survival data were extracted from our institutional electronic medical records system. Adjuvant chemotherapy regimens were selected at the investigator’s discretion from among four standard platinum-doublet options, administered in 21-day cycles.

### 2.2 Adjuvant treatment recommendations and ethical considerations

Adjuvant platinum-based chemotherapy was recommended for stage IB patients presenting with one or more high-risk features, including: poor tumor differentiation, visceral pleural invasion (VPI), sublobar resection, undetermined lymph node status (pNx), lymphovascular invasion (LVI), or spread through air spaces (STAS). Treatment allocation (ACT or observation) was determined through shared decision-making, considering each patient’s performance status (ECOG PS) and personal preferences. This study was conducted in compliance with Good Clinical Practice guidelines and the Declaration of Helsinki. The protocol was approved by the Ethics Committee of Chinese People’s Liberation Army General Hospital (Approval No. S2021-701-01). Written informed consent was obtained from all participants prior to study enrollment.

### 2.3 Follow-up

The follow-up protocol was conducted in accordance with the standardized procedures established in our prior investigation (17). Patient follow-up data were systematically collected through formal channels, including telephone interviews and comprehensive review of outpatient medical records, with the final follow-up conducted in May 2025. The primary study endpoint, DFS, was calculated as the duration (in months) from the surgical intervention to the occurrence of the first event (encompassing tumor recurrence, distant metastasis, or NSCLC-associated mortality) or the last documented follow-up for event-free patients. The secondary endpoint, OS, was defined as the time interval (measured in months) from the date of surgery to death from any cause.

### 2.4 Propensity score matching

To address potential selection bias resulting from baseline disparities between patients receiving ACT and those under clinical observation (CO), we employed propensity score matching (PSM) with careful consideration of clinically meaningful covariates (18). The matching protocol accounted for seven critical prognostic factors: patient demographics (age, sex), tumor morphology (maximum pathologic diameter), histologic grade (poor differentiation status), and three invasive pathological features (VPI, LVI, and STAS). Using a nearest-neighbor approach with a restricted caliper width (0.02 SD of the propensity score logit) and non-replacement sampling, we established balanced cohorts through 1:1 matching, thereby enhancing the reliability of subsequent comparative analyses while maintaining adequate statistical power.

### 2.5 Statistical analysis

Statistical analyses were conducted using SPSS 22.0 (IBM Corp., Armonk, NY) with rigorous attention to methodological appropriateness. Continuous variables were characterized using both parametric (mean ± standard deviation) and non-parametric (median, interquartile range) descriptors to accommodate different data distributions. For comparative analyses of continuous measures, the Mann-Whitney U test was employed to account for potential non-normal distributions. Categorical data were presented as absolute counts with corresponding percentages (n, %), with between-group comparisons performed using Pearson’s chi-square test with Yates’ continuity correction where appropriate. Time-to-event outcomes (DFS and OS) were analyzed through Kaplan-Meier survival estimation, with between-strata differences assessed via Mantel-Cox log-rank testing. All probability values were interpreted using a two-tailed α-level of 0.05 as the threshold for statistical significance.

## 3 Results

### 3.1 Clinical characteristics before and after PSM

Our institutional database identified 1130 consecutive stage IB NSCLC patients who underwent curative resection between January 2013 and August 2024. After applying stringent inclusion criteria, 181 patients formed the final study cohort (**Table 1**). The cohort comprised 50 patients (27.6%) who received ACT and 131 patients (72.4%) under CO. Pre-matching analysis revealed significant intergroup differences in several baseline characteristics. Patients in the ACT group demonstrated a younger age distribution (mean 59.6 ± 7.6 vs 63.8 ± 8.1 years, *P* = 0.002), with a higher proportion under 65 years (72.0% vs 49.6%, *P* = 0.007). Tumor morphology also differed substantially, with ACT cases presenting larger primary lesions (36.4 ± 3.7 mm vs 31.8 ± 7.3 mm, *P* < 0.001) and greater prevalence of tumors exceeding 3 cm (92.0% vs 61.8%, *P* < 0.001). Histopathological assessment showed poorer differentiation patterns more frequently in the ACT cohort (58.0% vs 35.9%, *P* = 0.017). These pretreatment imbalances were effectively mitigated through PSM, achieving balanced cohorts for subsequent comparative analyses.

**Table 1:** Patients’ clinical characteristics before and after PSM.

Characteristic	Entire Cohort		*P*	PSM Cohort		*P*
	ACT group	CO group		ACT group	CO group	
No.	50	131		46	46	
Sex			0.805			1
Male	43 (86)	115 (87.8)		39 (84.8)	39 (84.8)	
Female	7 (14)	16 (12.2)		7 (15.2)	7 (15.2)	
Age, years	59.56 ± 7.55	63.75 ± 8.12	0.002	60 ± 7.70	60.41 ± 8.96	0.813
<65	36 (72)	65 (49.6)	0.007	32 (69.6)	30 (65.2)	0.824
≥65	14 (28)	66 (50.4)	0.007	14 (30.4)	16 (34.8)	0.824
Smoking history	40 (80)	101 (77.1)	0.841	36 (78.3)	36 (78.3)	1
Family history of lung cancer	5 (10)	6 (4.6)	0.180	5 (10.9)	3 (6.5)	0.714
Abnormality of SCC	5 (10)	2 (3.2)	0.239	4 (8.7)	1 (3)	0.394
PET-CT	11 (10, 11.45)	10.8 (8.5, 14)	0.668	11 (10, 11.45)	10.8(8.5, 14)	0.953
Tumor location			0.270			0.391
RUL	15 (30)	36 (27.5)		13 (28.3)	8 (17.4)	
RML	3 (6)	9 (6.9)		3 (6.5)	4 (8.7)	
RLL	3 (6)	23 (17.6)		3 (6.5)	8 (17.4)	
LUL	13 (26)	35 (26.7)		13 (28.3)	15 (32.6)	
LLL	16 (32)	28 (21.4)		14 (30.4)	11 (23.9)	
Surgical procedure			1			0.315
sublobectomy	7 (14)	19 (14.5)		7 (15.2)	3 (6.5)	
lobectomy	40 (80)	105 (80.2)		36 (78.3)	41 (89.1)	
pneumonectomy	3 (6)	7 (5.3)		3 (6.5)	2 (4.3)	
LND						
Stations	4.80 ± 1.67	4.86 ± 1.81	0.832	4.78 ± 1.72	5.13 ± 1.47	0.301
Numbers	12 (7.5, 18)	7 (5, 14)	0.184	12 (7.5, 18)	7 (6, 14)	0.058
Tumor diameter(mm)	36.36 ± 3.73	31.79 ± 7.33	<0.001	36.09 ± 3.76	35.20 ± 5.25	0.352
>3cm	46 (92)	81 (61.8)	<0.001	42 (91.3)	38 (82.6)	0.354
≤3cm	4 (8)	50 (38.2)	<0.001	4 (8.7)	8 (17.4)	0.354
VPI	11 (22)	37 (28.2)	0.455	11 (23.9)	18 (39.1)	0.178
LVI	3 (6)	3 (2.3)	0.349	3 (6.5)	0	0.242
STAS	1 (2)	4 (3.1)	1.000	1 (2.2)	3 (6.5)	0.617
Ki-67 index	60 (57.5, 65)	70 (50, 70)	0.363	60 (57.5, 65)	70 (55, 70)	0.965
PD-L1	5 (2.5, 42.5)	20 (10, 50)	0.657	5 (2.5, 42.5)	20 (10, 50)	0.573
Cell differentiation			0.017			0.978
Well	1 (2)	1 (0.8)		1 (2.2)	1 (2.2)	
Moderate	20 (40)	83 (63.4)		20 (43.5)	21 (45.7)	
Poor	29 (58)	47 (35.9)		25 (54.3)	24 (52.2)	
Postoperative complications	1 (2)	6 (4.6)	0.675	1 (2.2)	6 (13)	0.111

Data presented as No. (%) unless otherwise noted.

PSM, propensity score matching; ACT, adjuvant chemotherapy; CO, clinical observation; SCC, squamous cell carcinoma antigen; PET-CT, positron emission tomography-computed tomography; RUL, right upper lobe; RML, right middle lobe; RLL, right lower lobe; LUL, left upper lobe; LLL, left lower lobe; CTR, consolidation tumor ratio; LND, lymph node dissection; VPI, visceral pleural invasion; LVI, lymphovascular invasion; STAS, spread through air spaces; PD-L1, programmed cell death-ligand 1.

### 3.2 Treatment patterns and tolerability of adjuvant chemotherapy regimens

The ACT cohort demonstrated distinct preferences in chemotherapy regimen selection, with cisplatin-docetaxel emerging as the most frequently prescribed combination (56%, n=28) (**Table 2**). Alternative regimens included cisplatin-gemcitabine (16%, n=8) and carboplatin-paclitaxel (28%, n=14). Notably, regimen completion rates varied substantially, with cisplatin-docetaxel achieving perfect adherence (100% completion) compared to 78.6% for carboplatin-paclitaxel. Treatment-related adverse events were common, affecting 80% (n=40) of ACT recipients. Toxicity profiles differed meaningfully across regimens: Cisplatin-gemcitabine demonstrated optimal tolerability with no grade ≥3 toxicities or treatment discontinuations, Carboplatin-paclitaxel showed intermediate toxicity but higher regimen incompletion rates, and Cisplatin-docetaxel, while having higher grade ≥3 events, maintained superior treatment completion. This pattern suggests that while platinum-doublet regimens exhibit class-effect toxicities, specific combinations may offer different risk-benefit profiles in the adjuvant setting for SqCC patients.

**Table 2:** Treatment patterns and tolerability of adjuvant chemotherapy regimens.

Distribution	Cisplatin + Docetaxel (n=28)	Cisplatin + Gemcitabine (n=8)	Carboplatin + Paclitaxel (n=14)
Duration			
2 cycles	0	1 (12.5)	3 (21.4)
4 cycles	28 (100)	7 (87.5)	11 (78.6)
Adverse events of any cause			
All	24 (85.7)	6 (75)	10 (71.4)
Leading to discontinuation of treatment	1 (3.6)	0	0
Grade ≥3	3 (10.7)	0	1 (7.1)

Data presented as No. (%) unless otherwise noted.

ACT, adjuvant chemotherapy.

### 3.3 Survival outcomes

**Figure 2:**
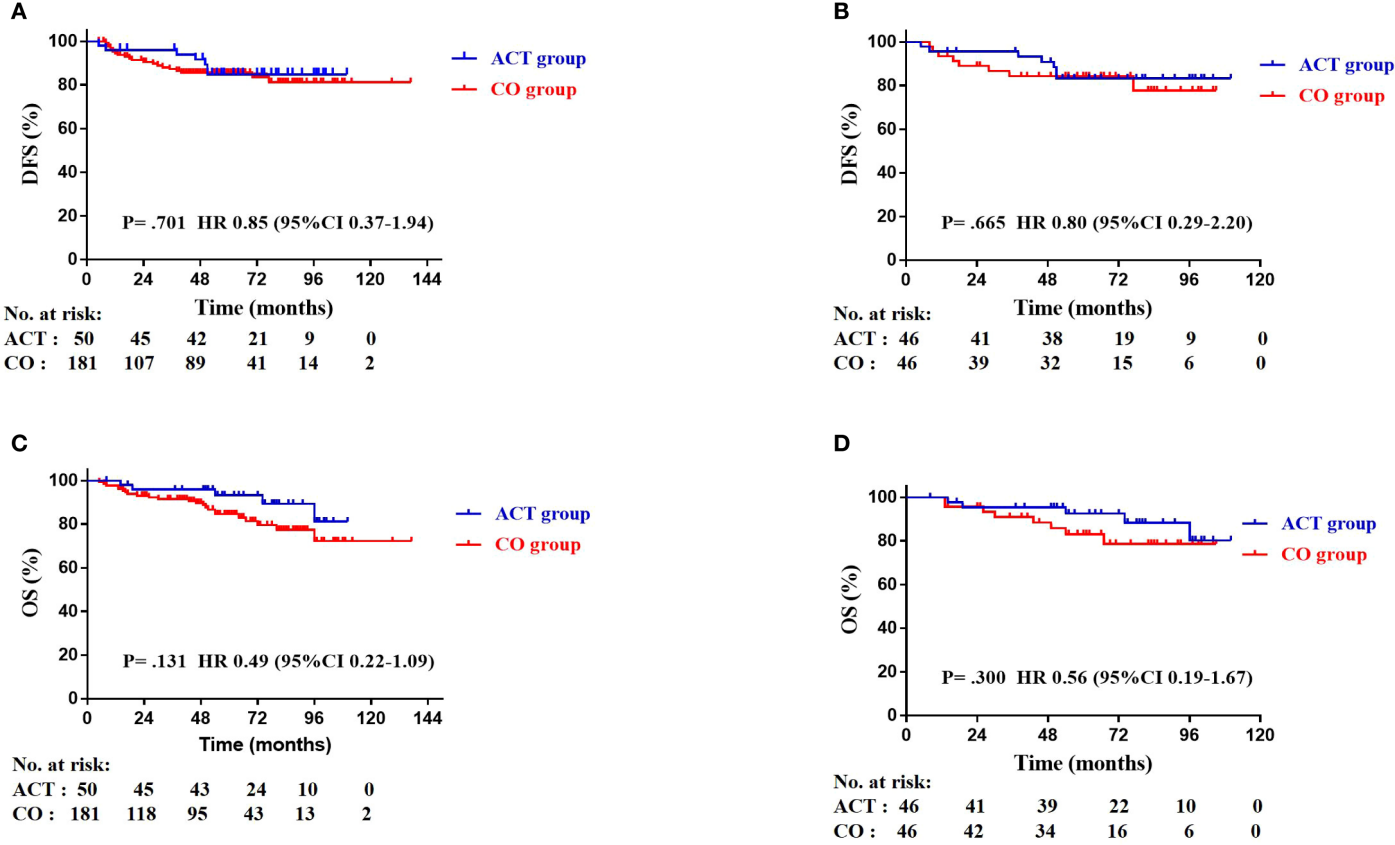
Kaplan-Meier plots of disease-free survival (DFS) in the entire cohort **(A)**, DFS in the matched cohort **(B)**, overall survival (OS) in the entire cohort **(C)**, and OS in the matched cohort **(D)**.

The median follow-up time was 60 months (range: 5–137 months). In the entire cohort, twenty-eight patients died and 27 patients had recurrence or metastasis at the last follow-up. Seven patients had recurrence or metastasis in the ACT group, three of them had local recurrence without distant metastasis, and four of them experienced both local recurrence and metastasis. Five of them died even with subsequent therapy. Twenty patients had recurrence or metastasis in the CO group, eight of them had local recurrence without distant metastasis, two of them only found out distant metastasis, and 10 patients experienced both local recurrence and metastasis. Thirteen patients died due to lung cancer-related cause. In the CO group, six patient died of myocardial infarction, one patient died of COVID-19, one patient died of cerebrovascular disease, and one patient died of multiple myeloma. Among the matched 92 patients, 7 (15.2%) patients in the ACT group and 8 (17.4%) patients in the CO group had disease relapse. The 3-year DFS rates were 93.8% in the ACT group and 87.3% in the CO group (HR: 3.12, 95% CI 0.72-13.56; *P* = 0.130). The 5-year DFS rates were 84.8% in the ACT group and 85.6% in the CO group (HR: 1.05, 95% CI 0.44-2.51; *P* = 0.919). There was no statistically significant difference in DFS between groups, both in the entire cohort (*P* = 0.701. **Figure 2A**) and the matched cohort (*P* = 0.665. **Figure 2B**). Five (10.9%) patients in the ACT group and 8 (17.4%) patients in the CO group died. The 5-year OS rates were 96.8% in the ACT group and 93.1% in the CO group (HR: 2.46, 95% CI 0.72-8.35; *P* = 0.150). Meanwhile, no statistically significant difference in OS was observed, either in the overall cohort (*P* = 0.131. **Figure 2C**) or in the matched cohort (*P* = 0.300. **Figure 2D**) between the two groups.

### 3.4 Analysis of DFS and OS factors

Univariate and subsequent multivariate analyses revealed that age, sex, tumor differentiation, tumor diameter, STAS, LVI, VPI, number of lymph node stations sampled, and ACT were not significantly associated with DFS (**Table 3**). Similarly, these factors did not demonstrate significant associations with OS in either univariate or multivariate analyses (**Table 4**).

**Table 3:** Univariate and multivariate Cox regression analysis of DFS factors.

DFS predictor	Univariate Analysis		Multivariate Analysis	
	HR (95% CI)	*P*	HR (95% CI)	*P*
Age	1.020 (0.958 to 1.086)	0.538	1.014 (0.949 to 1.084)	0.679
Sex	1.520 (0.428 to 5.402)	0.517	1.196 (0.306 to 4.685)	0.797
Tumor diameter	1.046 (0.919 to 1.190)	0.498	1.081 (0.932 to 1.255)	0.304
Tumor differentiation		0.997		0.962
High	–	0.987	–	0.992
Moderate	0.962 (0.348 to 2.660)	0.940	1.165 (0.399 to 3.403)	0.780
STAS	0.302 (0.039 to 2.364)	0.254	0.308 (0.025 to 3.757)	0.356
VPI	0.616 (0.219 to 1.734)	0.359	0.540 (0.166 to 1.757)	0.306
LVI	21.031 (0 to -)	0.665	–	0.990
Lymph node stations	0.900 (0.671 to 1.207)	0.481	0.890 (0.650 to 1.220)	0.469
ACT	1.250 (0.453 to 3.454)	0.666	1.149 (0.384 to 3.436)	0.804

DFS, disease-free survival; HR, hazard ratio; CI, confidential interval; STAS, spread through air spaces; VPI, visceral pleural invasion; LVI, lymphovascular invasion; ACT, adjuvant chemotherapy.

**Table 4:** Univariate and multivariate Cox regression analysis of OS factors.

OS predictor	Univariate Analysis		Multivariate Analysis	
	HR (95% CI)	*P*	HR (95% CI)	*P*
Age	1.032 (0.965 to 1.105)	0.358	0.997 (0.927 to 1.071)	0.928
Sex	1.556 (0.424 to 5.706)	0.505	1.011 (0.230 to 4.448)	0.988
Tumor differentiation		0.146		0.271
High	4.395 (0.523 to 36.919)	0.173	4.123 (0.352 to 48.275)	0.259
Moderate	0.492 (0.147 to 1.646)	0.250	0.526 (0.148 to 1.867)	0.320
Tumor diameter	0.980 (0.867 to 1.108)	0.751	1.008 (0.865 to 1.174)	0.918
STAS	0.193 (0.024 to 1.577)	0.125	0.222 (0.017 to 2.870)	0.249
LVI	20.993 (0 to 258463)	0.749	112780 (0 to -)	0.986
VPI	0.483 (0.162 to 1.442)	0.192	0.479 (0.136 to 1.683)	0.251
Lymph node stations	1.062 (0.750 to 1.503)	0.735	1.054 (0.715 to 1.555)	0.790
ACT	1.792 (0.584 to 5.500)	0.308	1.427 (0.426 to 4.774)	0.564

OS, overall survival; HR, hazard ratio; CI, confidential interval; STAS, spread through air spaces; VPI, visceral pleural invasion; LVI, lymphovascular invasion; ACT, adjuvant chemotherapy.

## 4 Discussion

This investigation represents one of the few contemporary real-world analyses evaluating adjuvant chemotherapy efficacy in stage IB squamous cell carcinoma (SqCC) using current TNM staging criteria, building upon the landmark CALGB 9633 trial (11) and subsequent work by Xu et al. (19). Our findings align with these previous studies in demonstrating no significant improvement in disease-free or overall survival with ACT compared to observation alone, thereby reinforcing the growing body of evidence questioning the therapeutic value of adjuvant platinum-based chemotherapy for this specific patient population. The consistent null results across multiple studies suggest the need to explore alternative adjuvant strategies for stage IB SqCC patients.

The current 9th edition TNM classification maintains consistency with its predecessor regarding T-stage criteria, preserving the 2017 modification that reclassified tumors measuring 4–5 cm from stage IB to IIA (3). Contemporary stage IB now encompasses tumors 3–4 cm in size or those demonstrating specific invasive features (visceral pleural involvement, main bronchus infiltration proximal to the carina, or hilar obstructive changes) (3). While platinum-based chemotherapy remains the standard adjuvant approach for stage II-IIIA disease, its role in stage IB continues to generate controversy despite advances in targeted therapies and immunotherapies (15, 20–22). Historical data from the IALT trial initially suggested potential benefits of cisplatin-based regimens in resected NSCLC (6), while the CALGB 9633 study specifically failed to demonstrate overall survival advantage in stage IB patients, though a non-significant trend emerged for larger tumors (≥4 cm) (11). These conflicting findings underscore the ongoing uncertainty surrounding adjuvant treatment decisions for this borderline tumor stage. Recent evidence regarding ACT efficacy in stage IB NSCLC remains conflicting. While the LACE meta-analysis established clear benefits for stage II-III disease, its findings for stage IB (T2N0) were inconclusive (23), potentially due to evolving TNM classification changes. Our analysis of stage IB SqCC revealed no significant DFS improvement with ACT (3-year: 93.8% vs 87.3%, *P* = 0.13; 5-year: 84.8% vs 85.6%, *P* = 0.919), nor any OS advantage. These results contrast with Xu et al.’s report (19) showing improved RFS (HR 0.58, *P* = 0.033) and OS (HR 0.49, *P* = 0.017) in 360 stage IB SqCC patients, particularly notable in their propensity-matched cohort. The observed discrepancies may stem from methodological differences, including their shorter median follow-up (45.9 vs our 60 months) and the apparent convergence of survival curves in their study over time. These conflicting findings highlight the need for further investigation into the durability of ACT benefits in stage IB SqCC and potential selection biases across studies.

Recent advances in adjuvant therapy have established distinct treatment paradigms for different NSCLC subtypes. For EGFR-mutant adenocarcinomas, landmark trials (ADAURA (15), CORIN (16), Shen et al. (17)) have validated EGFR-TKIs as standard adjuvant therapy, demonstrating significant DFS benefits. In SqCC, the PEARLS/KEYNOTE-091 trial (24) reported improved DFS with adjuvant pembrolizumab across stages IB-IIIA, though specific efficacy in stage IB (T≥4cm) remains unconfirmed. Notably, current immunotherapy trials have systematically excluded stage IB SqCC populations. While NCCN guidelines recommend considering adjuvant therapy (chemotherapy or osimertinib) for high-risk stage IB cases featuring poor differentiation, LVI, VPI, or suboptimal resection (14), our analysis of both overall and propensity-matched cohorts (20% with ≥1 risk factor) demonstrated no significant DFS (*P* = 0.919) or OS improvement with ACT. Further univariate and multivariate Cox regression analyses demonstrated no significant association between tumor differentiation, STAS, LVI, VPI, or ACT and either DFS or OS.These findings question the universal applicability of current high-risk criteria for adjuvant chemotherapy decisions in stage IB SqCC, particularly in light of emerging immunotherapy options that may offer alternative strategies for this patient population. Emerging evidence suggests traditional clinicopathological risk factors may be insufficient for predicting adjuvant chemotherapy benefit in stage IB NSCLC. Recent studies have identified circulating tumor DNA (ctDNA) as a promising biomarker for minimal residual disease detection, with multiple trials demonstrating its strong correlation with disease recurrence in stages I-IIIA NSCLC (25–27). While our study could not incorporate ctDNA analysis due to financial constraints, compelling data from landmark trials support its clinical utility. The ADAURA trial showed osimertinib achieved 72% ctDNA clearance in EGFR-mutant patients, correlating with significantly improved DFS (HR = 0.38) (15). Similarly, IMpower010’s exploratory analysis suggested enhanced atezolizumab efficacy in PD-L1-positive, ctDNA-positive cases (HR = 0.42) (21), though these findings require confirmation. For stage IB squamous cell carcinoma with high-risk features, postoperative ctDNA monitoring may offer superior risk stratification compared to conventional parameters, potentially enabling more personalized adjuvant therapy decisions by identifying patients most likely to benefit from systemic treatment.

Several limitations should be considered when interpreting our findings. First, the retrospective design introduces potential selection bias and limits causal inference, though we employed propensity score matching to mitigate baseline characteristic imbalances. Second, the relatively small sample size may reduce statistical power to detect modest treatment effects, highlighting the need for larger prospective trials. Moreover, the heterogeneity of chemotherapy regimens and the lack of detailed balance diagnostics post-PSM also weaken the validity of the results. Fourth, the lack of minimal residual disease assessment through circulating tumor DNA (ctDNA) analysis represents a significant constraint, as emerging evidence strongly supports its prognostic value and potential for guiding adjuvant therapy decisions in early-stage NSCLC. Our ongoing registered multicenter prospective study will provide more definitive evidence regarding optimal adjuvant strategies for stage IB squamous cell carcinoma.

## 5 Conclusions

Our findings suggest adjuvant platinum-based chemotherapy may not confer significant survival benefits in resected stage IB squamous cell carcinoma, with comparable disease-free and overall survival outcomes observed between treatment and observation groups.

## Data Availability

The raw data supporting the conclusions of this article will be made available by the authors, without undue reservation.
